# Maternal acellular pertussis vaccination in mice impairs cellular immunity to *Bordetella pertussis* infection in offspring

**DOI:** 10.1172/jci.insight.167210

**Published:** 2023-09-22

**Authors:** Violaine Dubois, Jonathan Chatagnon, Manon Depessemier, Camille Locht

**Affiliations:** University Lille, CNRS, Inserm, CHU Lille, Institut Pasteur de Lille, U1019 – UMR9017 – CIIL – Center for Infection and Immunity of Lille, Lille, France.

**Keywords:** Infectious disease, Vaccines, Bacterial vaccines, Cellular immune response, Mouse models

## Abstract

Given the resurgence of pertussis, several countries have introduced maternal tetanus, diphtheria, and acellular pertussis (aP) vaccination during pregnancy to protect young infants against severe pertussis. Although protective against the disease, the effect of maternal aP vaccination on bacterial colonization of the offspring is unknown. Here, we used a mouse model to demonstrate that maternal aP immunization, either before or during pregnancy, protects pups from lung colonization by *Bordetella pertussis*. However, maternal aP vaccination resulted in significantly prolonged nasal carriage of *B. pertussis* by inhibiting the natural recruitment of IL-17–producing resident memory T cells and ensuing neutrophil influx in the nasal tissue, especially of those with proinflammatory and cytotoxic properties. Prolonged nasal carriage after aP vaccination is due to IL-4 signaling, as prolonged nasal carriage is abolished in IL-4Rα^–/–^ mice. The effect of maternal aP vaccination can be transferred transplacentally to the offspring or via breastfeeding and is long-lasting, as it persists into adulthood. Maternal aP vaccination may, thus, augment the *B*. *pertussis* reservoir.

## Graphical abstract

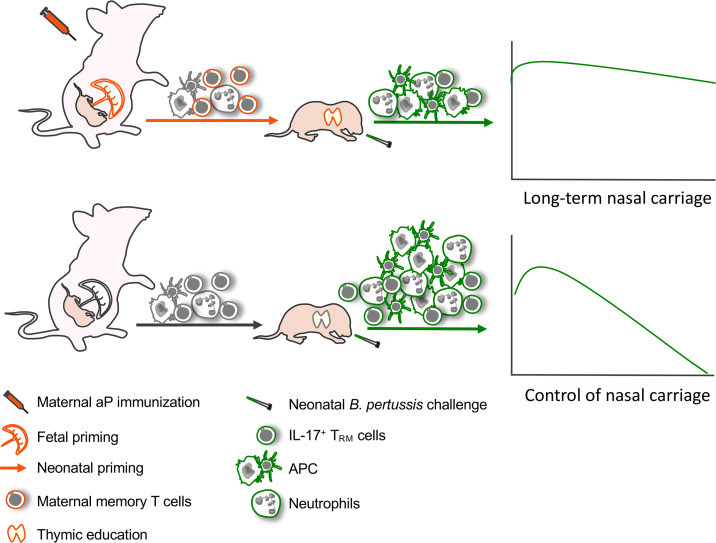

## Introduction

Pertussis, mainly caused by *Bordetella pertussis*, is a severe respiratory disease that is reemerging in several areas of the world, despite high vaccination coverage (1, 2). The highest attack and pertussis-related mortality rates are consistently seen in young infants who are too young to be vaccinated or have not completed their primary immunization series (3, 4). Cocoon vaccination with tetanus, reduced-dose diphtheria, acellular pertussis (Tdap) vaccine has been proposed but is difficult to implement (5) and did in fact not limit the number of pertussis cases in the very young (6, 7). As an alternative to protecting young infants against severe pertussis, maternal pertussis immunization has been implemented in several countries. Vaccination during pregnancy boosts the concentration of maternal vaccine–specific antibodies and the quantity transported to the fetus in utero (8). Maternal antibodies can effectively neutralize pertussis toxin in *B*. *pertussis*–infected neonates, and this is sufficient to prevent the symptoms of pertussis (9, 10). Maternal Tdap immunization provides thereby protection against severe and life-threatening clinical pertussis during the first months of life (11–13).

Although neonates have an inexperienced immune system, mainly polarized toward the Th2 type (14, 15), the early life immune system is not in a fixed state of immaturity and rapidly adapts to environmental cues. Murine and human neonatal CD4^+^ and CD8^+^ T cells can mount adult-like responses when stimulated in suitable conditions, such as by appropriate adjuvants (16), DNA immunization (17), low doses of viruses (18), or adult antigen-presenting cells (APCs) (19). There is also evidence that the fetal immune system may be influenced as a result of maternal vaccination by more than just passive immunity provided through transplacental IgG transfer. In utero priming has been suggested in animal studies (20) and in studies of uninfected children born to mothers infected with HIV (21–24). In addition, the fetal immune system may be sensitized in utero to vaccine antigens to which the mother has been exposed during pregnancy (25). Infant immune programming during breastfeeding has also been evidenced in animal and limited human-based studies (26).

We (27) and others (28) have shown that acellular pertussis (aP) vaccination substantially prolongs *B*. *pertussis* carriage in the noses of Th2-prone adult BALB/c mice. Similar observations have also been made in nonhuman primates (29). In adult mice, prolonged carriage was shown to be due to the inhibition by aP vaccines of naturally induced recruitment of IL-17–producing resident memory CD4^+^ T (Trm) cells and the ensuing neutrophils in the nasal tissue (27). In this study, we used the mouse model to examine the effect of maternal aP immunization on the natural induction of Th17 cells and neutrophils and the resulting nasal clearance of *B*. *pertussis* in the offspring.

## Results

### Maternal aP immunization prolongs nasal carriage of B. pertussis in infected neonatal mice.

**Figure 1:**
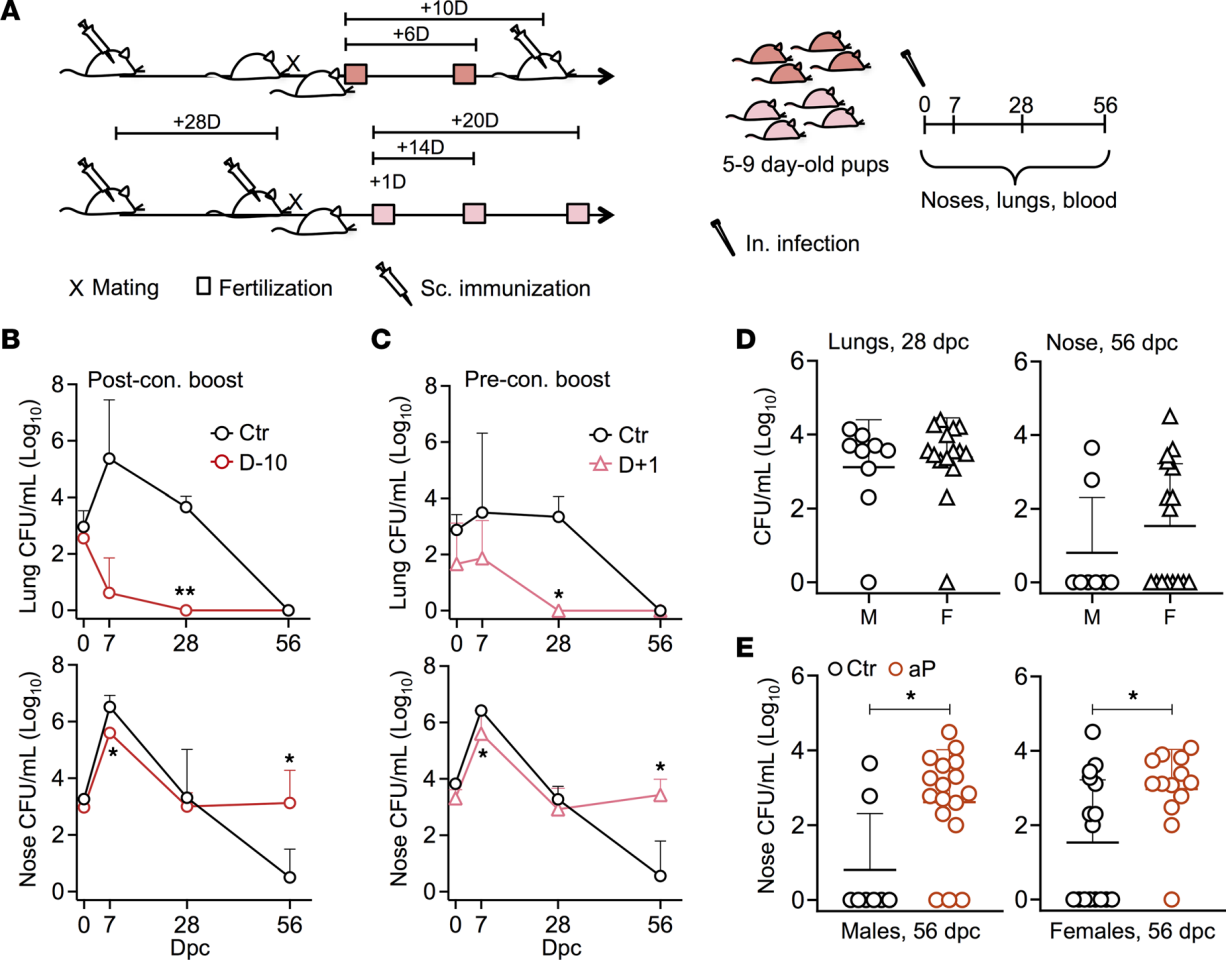
Antenatal aP immunization prevents lung colonization in offspring while enhancing nasal carriage. (**A**) Female BALB/c mice were immunized twice s.c. with 1/10 human dose of Infanrix (aP). Control mice received PBS. Mice were mated 28 days after priming and boosted on the day of the mating (preconception [pre-con.] boost) or 10 days later (post-con. boost). For post-con. boosting, fertilization occurred at the day of mating — i.e., 10 days before boosting (D–10) — or 6 days after mating — i.e., 4 days before boosting (D–4). For the preconception boosting, fertilization occurred 1 (D+1), 14 (D+14), or 20 (D+20) after boosting. Five to 9 days after birth, the pups were nasally infected with 5 × 10^3^ CFU B1917. At the indicated time points after challenge, lungs and noses were harvested for CFU counting and blood was collected to measure maternally transferred anti–*B*. *pertussis* IgG. (**B**) CFU counts in lungs (upper panel) and noses (lower panel) from pups born to mothers boosted 10 days after fertilization (D–10) compared with control mice (Ctr) at indicated time points after challenge. (**C**) CFU counts in lungs (upper panel) and noses (lower panel) from pups born to mothers boosted 1 day before fertilization (D+1) compared with control mice (Ctr) at indicated time points after challenge. (**D**) CFU counts in lungs (left panel) and noses (right panel) of male (M) and female (F) pups born to nonvaccinated mothers 28 days (left panel) or 56 days (right panel) after challenge. (**E**) CFU counts in the noses of male (left panel) and female (right panel) pups born to nonvaccinated (Ctr) or aP-vaccinated (aP) mothers 56 days after challenge. Results shown are geometric means ± SD. *n* = 3–6 for the Ctr groups and *n* = 3–5 for the aP groups in (**B** and **C**). Mann-Whitney tests were performed to compare Ctr and aP offspring. *P < 0.05; **P < 0.01.

To investigate the influence of maternal aP immunization on *B*. *pertussis* infection of the offspring, adult female BALB/c mice were immunized s.c. with a 1/10 human dose of aP 28 days prior to mating. Control mice received phosphate-buffered saline (PBS) instead. Fertilization occurred at the day of mating or 6 days later. Ten days after mating, the mice were boosted once (Figure 1A, upper left). The neonatal (5- to 9-day-old) mice were then nasally challenged with 5 × 10^3^ colony-forming units (CFU) of *B*. *pertussis* B1917. Lungs and noses were harvested at various time points after challenge for CFU counting (Figure 1A, right). Blood was also collected to detect maternally transferred *B*. *pertussis*–specific IgG by ELISA (Figure 1A, right).

In the control offspring, the bacterial load in the lungs reached 1 ***×*** 10^6^ CFU 7 days postchallenge (dpc) and then decreased slowly until complete clearance 56 dpc. Maternal aP immunization protected the offspring against lung infection by *B*. *pertussis*, regardless of the time of boosting during pregnancy (Figure 1B and Supplemental Figure 1A; supplemental material available online with this article; https://doi.org/10.1172/jci.insight.167210DS1). In contrast to the protective effect against lung *B*. *pertussis* colonization in the offspring, maternal aP vaccination did not protect the pups against nasal colonization by B1917 (Figure 1B and Supplemental Figure 1A). In the control pups, the bacterial load in the nose reached 1 ***×*** 10^7^ CFU 7 dpc and then gradually decreased, reaching the detection limit at 56 dpc. In contrast, neonates born to aP-vaccinated mice still carried substantial amounts of bacteria (1 ***×*** 10^2^ to 1 ***×*** 10^4^ CFU) 56 dpc (Figure 1B and Supplemental Figure 1A).

We performed similar experiments to examine the role of boosting before fertilization on *B*. *pertussis* infection of the offspring. Twenty-eight days after the first vaccination, mice received an aP booster dose, followed by mating. Fertilization occurred 1, 14, or 20 days later (Figure 1A, lower left). The pups were nasally challenged with 5 × 10^3^ CFU of *B*. *pertussis* B1917 5–9 days after birth. Boosting before pregnancy also resulted in protection against lung colonization (Figure 1C and Supplemental Figure 1B) but substantially prolonged nasal carriage of *B*. *pertussis* in the pups (Figure 1C and Supplemental Figure 1B), although in this experiment, when fertilization occurred at 20 days after boosting, the differences between the aP and control mice did not reach statistical significance. Prolonged nasal carriage in the offspring was also observed when the mothers were boosted at least 2.5 months before fertilization (Supplemental Figure 2). To determine whether sex may affect the lung or nasal colonization by *B*. *pertussis*, we separately analyzed the male and female offspring of vaccinated and nonvaccinated mothers. While there was no significant impact of sex on lung colonization (Figure 1D), female pups from nonvaccinated mothers tended to carry the bacteria longer in the nose than male pups (Figure 1D). However, the effect of maternal aP immunization on nasal carriage of the offspring did not differ between male and female pups (Figure 1E).

### Placental and breast milk–derived inhibition of nasal B. pertussis clearance in the offspring.

**Figure 2:**
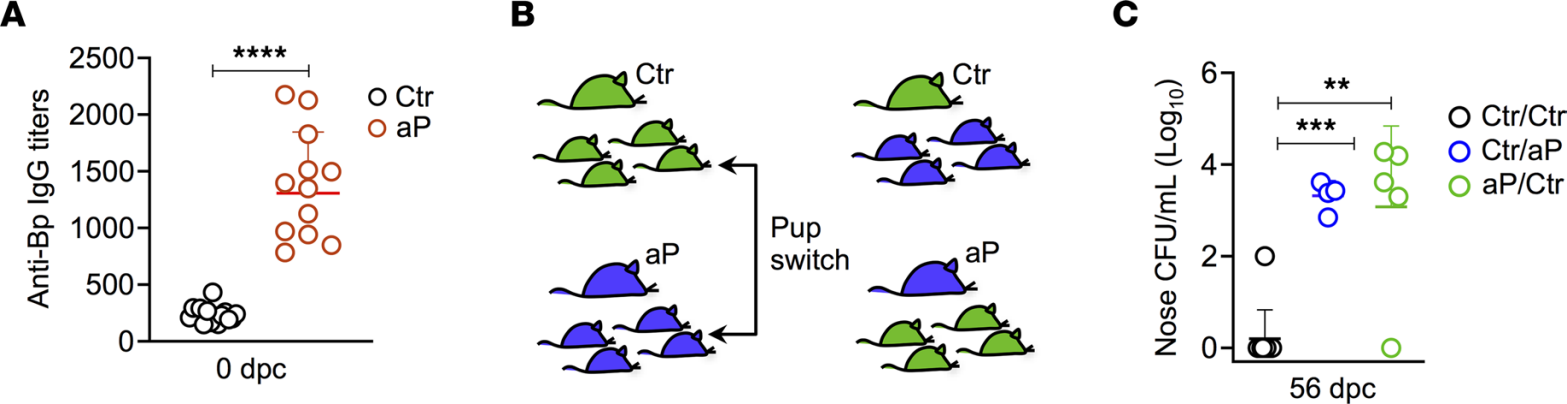
Placental and breast milk–derived inhibition of nasal *B*. *pertussis* clearance in the offspring. (**A**) Anti–*B*. *pertussis* IgG titers in the serum of control (Ctr) and aP offspring 3 hours after infection (*n* = 12–15). (**B** and **C**) Effect of nursing on nasal colonization of the offspring. Offspring from control (Ctr, in green) and aP-vaccinated mothers (aP, in blue) were switched within 12 hours after birth. Control pups nursed by aP-immunized mice (Ctr/aP) and pups from aP-vaccinated mothers nursed by control dams (aP/Ctr) were infected with 5 × 10^3^ CFU B1917 and sacrificed 56 dpc to assess the bacterial burden in the noses. Data on Ctr pups (Ctr/Ctr) are derived from the results shown in Figure 1 (*n* = 4–19). Mann-Whitney tests were performed to compare Ctr/Ctr with Ctr/aP or aP/Ctr offspring. **P < 0.01; ***P < 0.005; ****P < 0.001.

Pups born to aP-immunized mice had high amounts of maternally derived circulating IgG against *B*. *pertussis* (Figure 2A), suggesting that maternal antibodies participated in the prevention of lung colonization in the offspring, a finding consistent with previous observations showing that protection against lung colonization was mediated by both transplacentally acquired antibodies and breastfeeding (30). We were unable to perform nasal washes of the neonatal mice due to their small size, and this prevented us from determining *B*. *pertussis*–specific IgG or IgA levels in their nasal cavity.

To determine whether the negative effect of maternal aP immunization on nasal colonization of the offspring occurred through the transplacental or the breast-feeding route, we switched pups born to control or aP-immunized mice immediately after birth to aP-vaccinated and control mothers, respectively (Figure 2B). As shown in Figure 2C, compared with control pups born to control mothers, persistent nasal carriage of *B*. *pertussis* was observed after switching in both groups of foster pups, suggesting that both breastfeeding and in utero imprinting influence the pups’ ability to control nasal infection by *B*. *pertussis*.

### Maternal aP immunization is associated with impaired recruitment and activation of Th cells in the lower and upper respiratory tracts of infected offspring.

**Figure 3:**
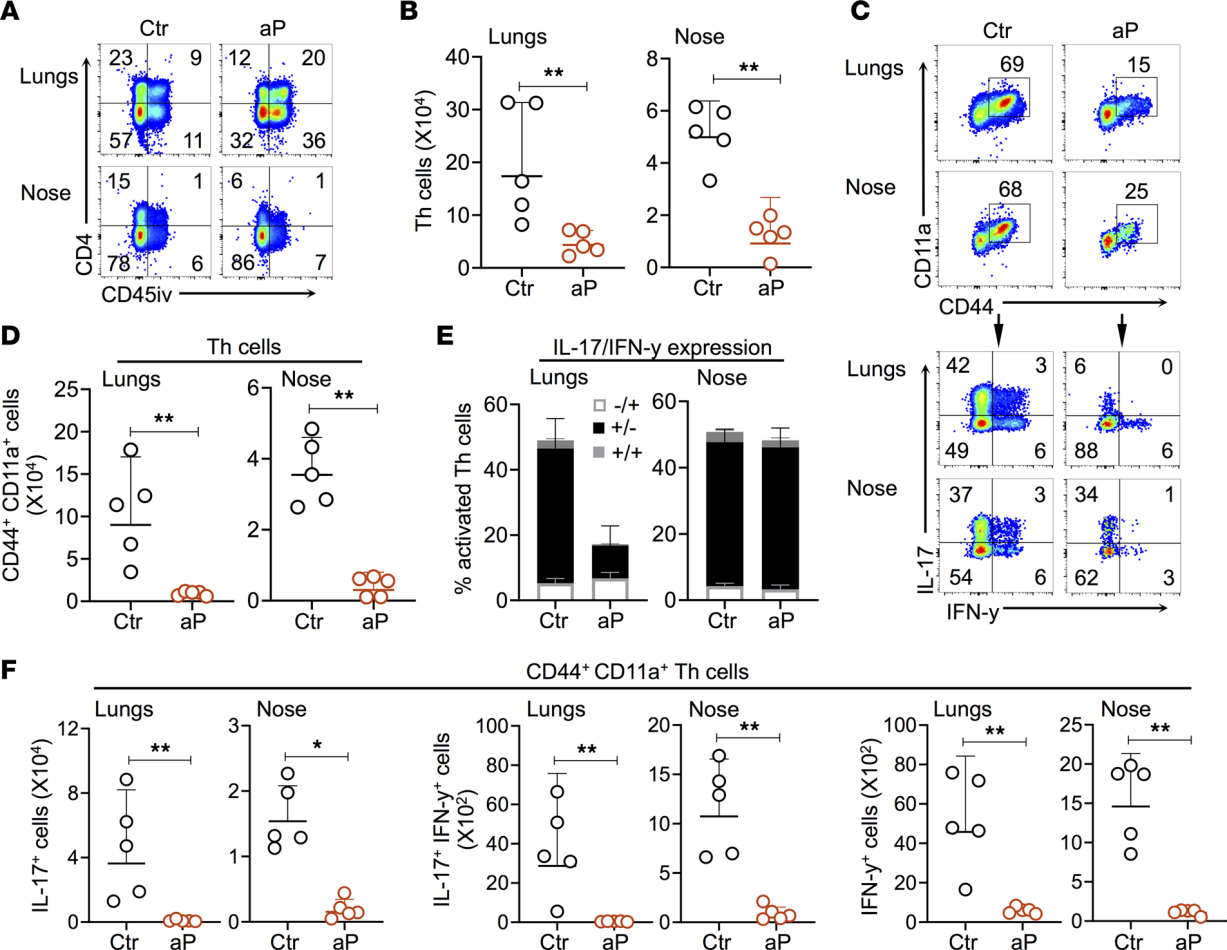
Effect of maternal aP immunization on recruitment and activation of T cells in lungs and noses. Neonatal mice born to control (Ctr) or aP-vaccinated mothers (aP) were infected with 5 × 10^3^ CFU B1917 6–7 days after birth and sacrificed 28 dpc. Ten minutes before euthanasia, mice were i.v. injected with anti–CD45-PE (CD45iv) antibody. (**A**) Representative plots showing the recruitment of CD4^+^CD45iv^–^ T cells in the lungs (upper plots) and noses (lower plots). (**B**) Absolute numbers of CD4^+^CD45iv^–^ T cells in the lungs (left panel) and nose (right panel) of offspring. (**C**) Representative plots showing the expression of CD11a and CD44 on CD4^+^ T cells (upper panel) and expression of IL-17 and IFN-γ upon stimulation with PMA and ionomycin (lower panel). (**D**) Absolute numbers of pulmonary (left) and nasal (right) CD4^+^ T cells expressing CD44 and CD11a. (**E**) Percentages of CD4^+^ T cells producing IL-17 and/or IFN-γ in lungs (left) and noses (right). (**F**) Absolute numbers of CD44^+^CD11a^+^CD4^+^ T cells expressing IL-17 (left panel), IFN-γ (right panel), or both (middle panel). Results shown are geometric means ± SD. *n* = 4–5. Mann-Whitney tests were performed to compare Ctr and aP offspring. *P < 0.05; **P < 0.01.

Several studies have shown that, in adult mice, local IL-17–producing CD4^+^ Trm cell responses are required to efficiently clear *B*. *pertussis* from the upper respiratory tract (27, 31). We therefore investigated the effect of maternal aP immunization on the recruitment of CD4^+^ Trm cells upon *B*. *pertussis* infection in the lung and nasal tissues of the offspring. To identify these cells in the nasal and lung tissues, mice were injected i.v. with an anti–CD45-PE antibody (CD45iv), enabling us to distinguish infiltrated resident cells from circulating immune cells. Recruitment of CD4^+^CD45iv^–^ T cells to the lungs and noses of offspring from aP-vaccinated mothers was significantly reduced 28 dpc with *B*. *pertussis* compared with control mice (Figure 3, A and B). The amounts of CD4^+^CD45iv^–^ T cells expressing the activation markers CD44 and CD11a were also diminished in both tissues of pups from aP-vaccinated mothers (Figure 3, C and D). Upon stimulation with phorbol myristate (PMA) and ionomycin, less than 20% of lung Th cells from the aP pups produced IFN-γ and/or IL-17, compared with approximately 50% of the control pups, in contrast to the equal proportions (50%) of IL-17–producing Th cells in the nasal tissue (Figure 3, C and E). Nonetheless, the amount of CD4^+^ T cells expressing IL-17, IFN-γ, or both IL-17 and IFN-γ was substantially reduced in both the lungs and the noses of offspring from vaccinated mothers (Figure 3F). Although only a minor proportion of Trm cells, CD8^+^ T cells also showed decreased amounts of CD44^+^CD11a^+^ cells in lungs and noses, as well as diminished numbers of IL-17– and/or IFN-γ–producing cells (Supplemental Figure 3).

**Figure 4:**
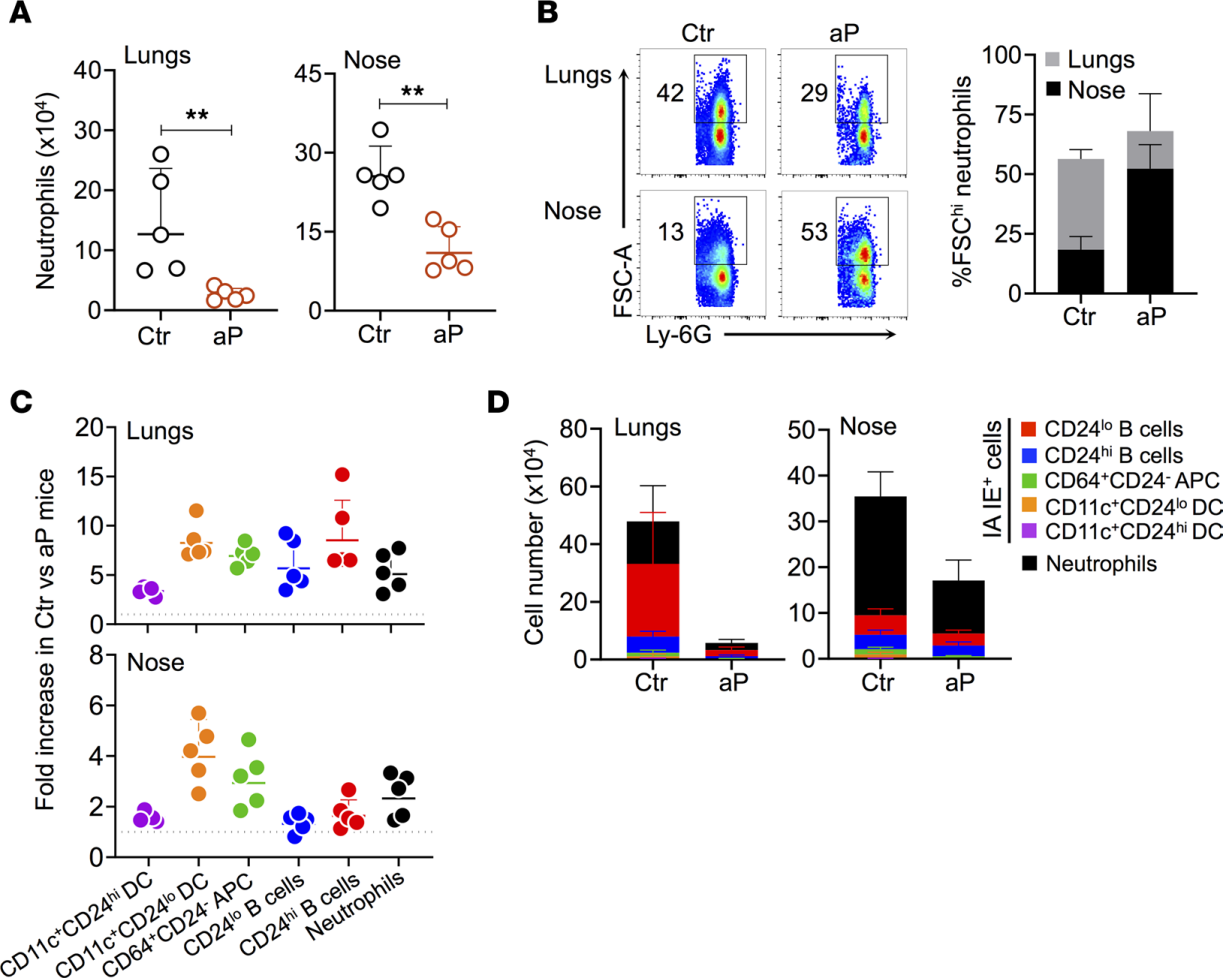
Effect of maternal aP immunization on recruitment of neutrophils and phagocytic cells in lungs and noses of the offspring. Neonatal mice born to control (Ctr) or aP-vaccinated mothers (aP) were infected with 5 ***×*** 10^3^ CFU B1917 6– 7 days after birth and sacrificed 28 dpc. Ten minutes before euthanasia, mice were i.v. injected with anti–CD45-PE antibody (CD45iv). (**A**) Absolute numbers of neutrophils in lungs and noses. (**B**) Representative plots showing high–FSC-A (FSC^hi^) Ly-6G^+^ neutrophils in lungs and noses (left panel) and corresponding percentages (right panel). (**C**) Fold increases of phagocytic cells with the indicated phenotype in Ctr over aP offspring in lungs (upper panel) and noses (lower panel). (**D**) Percentages of phagocytic cells with the indicated phenotypes in lungs (left) and noses (right). Results shown are geometric means ± SD. *n* = 4–5. Mann-Whitney tests were performed to compare Ctr and aP offspring. **P < 0.01.

To determine whether the decrease in Th cell recruitment and activation in the lungs and noses of aP pups was associated with differences in neutrophil recruitment, we determined the amounts of neutrophils recruited in lungs and noses 28 dpc in aP and control pups. Neutrophil influx was substantially decreased in the lungs and noses of aP offspring (Figure 4A).

Neutrophils can be divided into 2 subtypes, PMN-I, which are small (FSC^lo^) with marked proinflammatory and cytotoxic properties, and PMN-II, which are larger (FSC^hi^) and display antiinflammatory properties, characterized by the expression of IL-10 (32). The proportion of FSC^hi^ neutrophils was strongly augmented in the noses of aP offspring compared with the controls, while this was not the case in the lungs (Figure 4B). Thus, maternal aP immunization alters neutrophil numbers and function in the nasal mucosa of the offspring. We have previously shown that the impairment of neutrophil recruitment in the nasal cavity was associated with prolonged nasal carriage. To confirm that neutrophils are involved in nasal clearance of *B*. *pertussis*, we treated the mice with anti–Ly-6G antibodies 1 day before challenge and every 2 days after challenge, and we compared nasal CFU counts to those of infected mice treated with an isotype-control antibody. While the CFU counts in the noses were similar between the 2 groups 3 dpc, anti–Ly-6G antibody-treated mice carried significantly more bacteria in the noses than the control mice 14 dpc (Supplemental Figure 4).

We also determined the effect of maternal aP vaccination on APCs in lungs and noses of the offspring, including B cells and DCs. All analyzed cell populations were reduced in the noses and lungs of aP offspring 28 dpc (Figure 4C) — in particular, CD11c^+^CD24^lo^ DCs, CD64^+^CD24^–^ APC, and CD24^lo^ B cells (5-fold or more) in the lungs (Figure 4C). However, the cellular composition in lung and nose tissues remained unchanged (Figure 4D). Mature B cells (CD24^lo^) predominated over neutrophils in the lungs, while neutrophils prevailed in the noses (Figure 4D).

### Maternal aP immunization also alters offspring Th balance in secondary lymphoid tissues.

**Figure 5:**
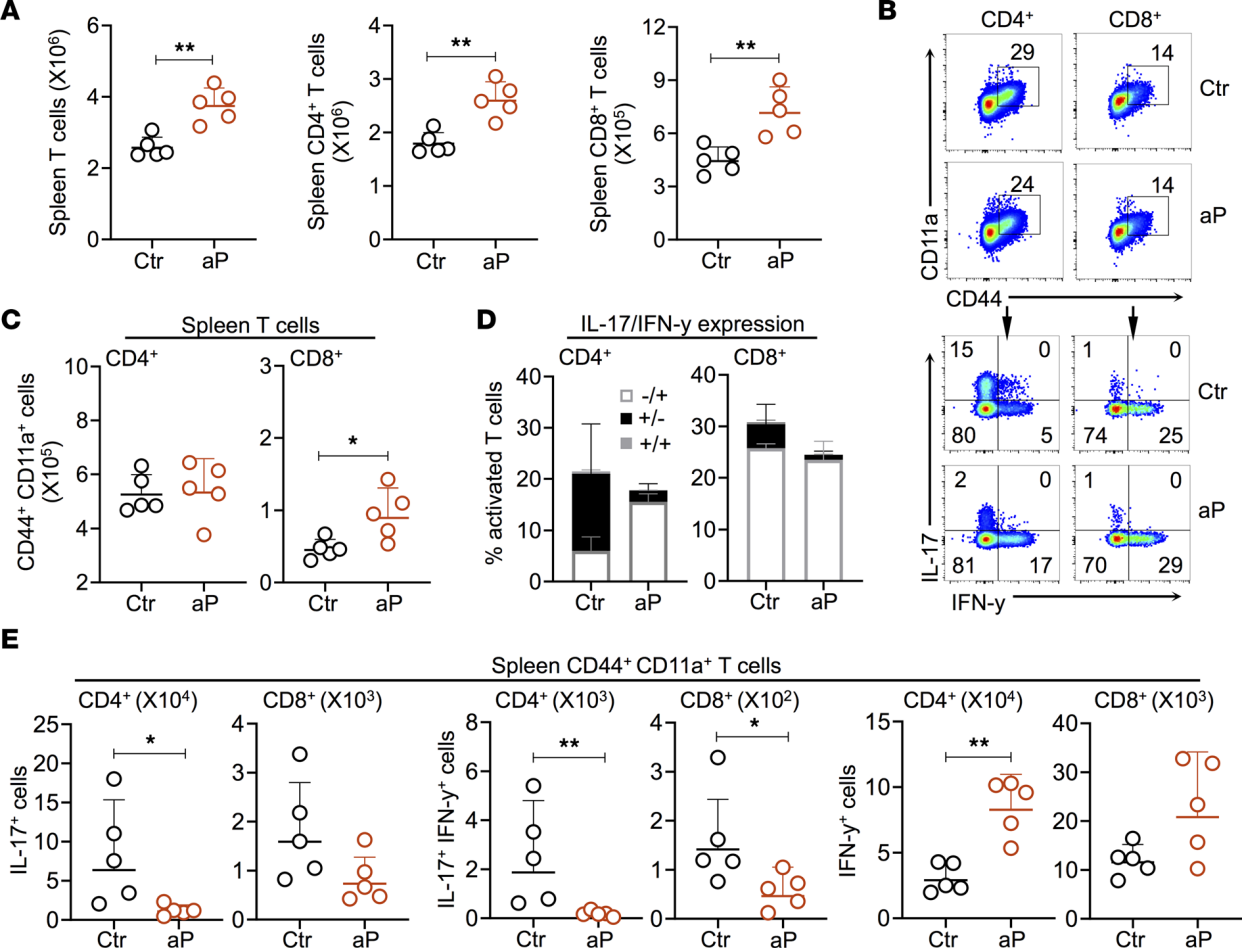
Effect of maternal aP immunization on the natural induction of Th17 cells in secondary lymphoid tissues. (**A**) Absolute numbers of T cells (left panel), CD4^+^ (middle panel), and CD8^+^ T cells in the spleen of aP (red dots) and control (black dots) offspring 28 dpc. (**B**) Representative plots showing the expression of CD11a and CD44 on splenic T cells (upper panel) and expression of IL-17 and IFN-γ upon stimulation with PMA and ionomycin (lower panel) by splenic T cells from pups born to control (Ctr) or aP-vaccinated (aP) mothers. (**C**) Absolute numbers of splenic CD4^+^ (left panel) and CD8^+^ T cells expressing CD44 and CD11a from pups born to control (Ctr) or aP-vaccinated (aP) mothers. (**D**) Percentages of activated splenic CD4^+^ (left panel) and CD8^+^ (right panel) T cells from pups born to control (Ctr) or aP-vaccinated (aP) mothers producing IL-17 and/or IFN-γ. (**E**) Absolute numbers of splenic CD44^+^CD11a^+^CD4^+^ and CD44^+^CD11a^+^CD8^+^ T cells expressing IL-17 (left panel), IFN-γ (right panel), or both (middle panel). Results shown are geometric means ± SD. *n* = 5. Mann-Whitney tests were performed to compare Ctr and aP offspring. *P < 0.05; **P < 0.01.

The influence of maternal aP immunization on the cellular immune responses of the offspring upon *B*. *pertussis* infection is not limited to the mucosal T cell responses. However, in contrast to the mucosal T cell responses, T cell responses in the spleen of aP offspring 28 days after challenge were stronger than in the control mice, with respect to both CD4^+^ and CD8^+^ T cells (Figure 5A). However, the amounts of CD4^+^ T cells expressing both CD11a and CD44 did not differ between the 2 groups of mice (Figure 5, B and C), while the numbers of CD8^+^CD44^+^CD11a^+^ T cells were slightly increased in aP offspring (Figure 5, B and C). Both CD4^+^CD44^+^CD11a^+^ and CD8^+^CD44^+^CD11a^+^ T cells were able to secrete IFN-γ and/or IL-17 upon ex vivo stimulation with PMA and ionomycin (Figure 5, B, D, and E). However, compared with the control offspring, the aP offspring showed reduced IL-17 and IL-17 + IFN-γ production by CD4^+^CD44^+^CD11a^+^ and CD8^+^CD44^+^CD11a^+^ T cells, while we observed twice as many IFN-γ^+^ T cells in the spleen of aP offspring compared with the controls (Figure 5, B, D, and E). Thus, maternal aP immunization also prevented the natural induction of Th17 cells in secondary lymphoid tissues in the offspring.

### Prolonged nasal carriage of B. pertussis in aP-immunized BALB/c mice and aP offspring depends on IL-4 signaling.

**Figure 6:**
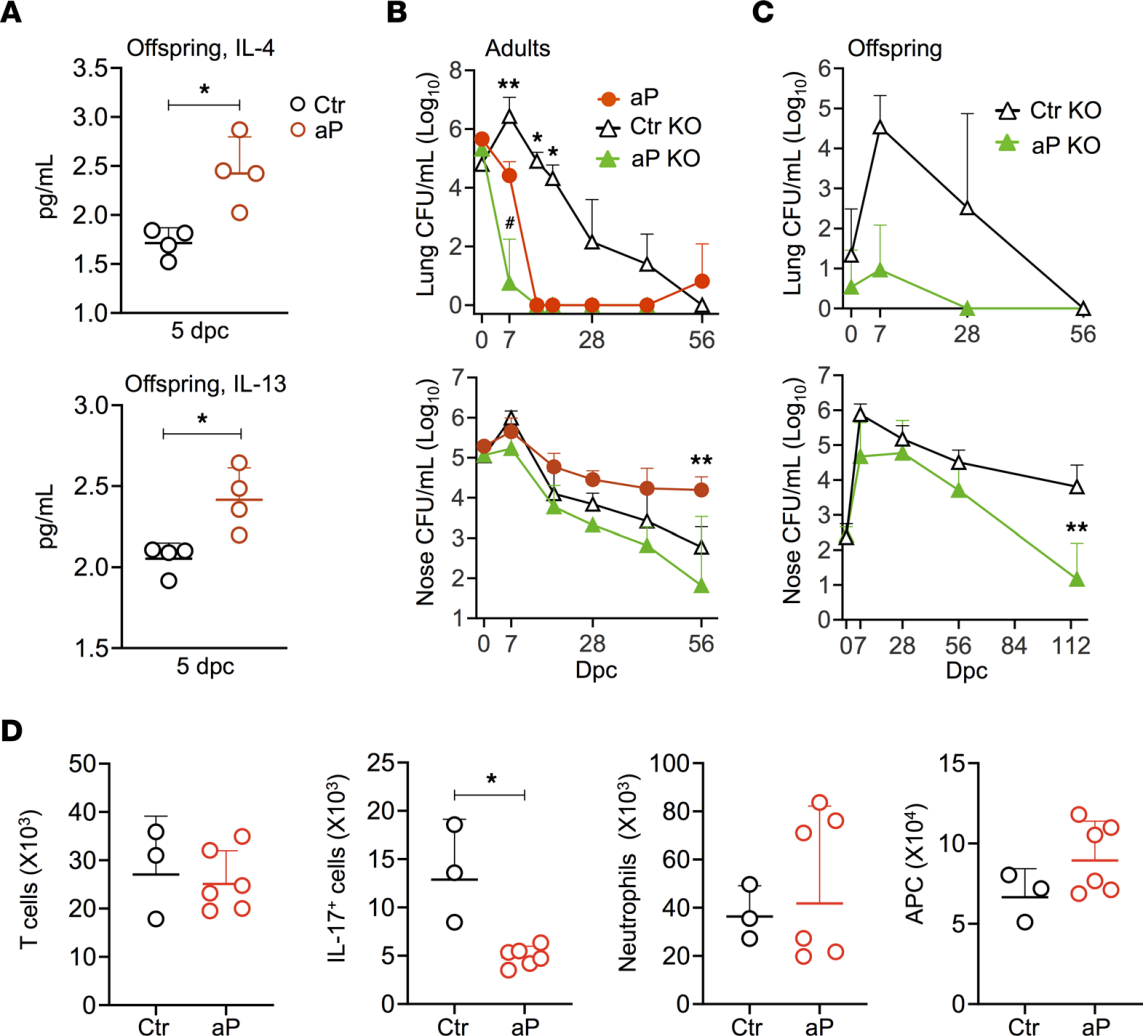
Role of the IL-4 pathway in prolonged nasal *B*. *pertussis* carriage in aP-immunized BALB/c mice. (**A**) Concentrations of IL-4 and IL-13 in the serum of neonates born to aP-immunized (aP) or control mice (Ctr) 5 days after challenge. (**B** and **C**) Six- to 8 week-old BALB/c and IL-4Ra^–/–^ (KO) mice, were immunized twice s.c. with 1/10 human dose of aP or PBS and then challenged with 1 ***×*** 10^6^ CFU B1917 (**B**). Neonates were nasally infected with 5 × 10^3^ CFU B1917 (**C**). Bacterial burden was determined by CFU counting on homogenized lungs (upper panels) and noses (lower panels) at indicated time points after challenge. (**D**) Absolute numbers of T cells (left panel), IL-17^+^ T cells (middle left panel), neutrophils (middle right panel), and APCs (right panel) in the noses of aP (red dots) and control (black dots) IL-4Rα^–/–^ mice 28 dpc. Results shown are geometric means ± SD. *n* = 5. Mann-Whitney tests were performed to compare Ctr and aP offspring and control KO with aP mice and aP KO mice. *P < 0.05; **P < 0.01.

Human and murine neonatal immune responses are polarized mainly toward the Th2 type (14, 15), and aP vaccination preferentially induces Th2 cells in adult mice and in human infants (33, 34). IL-4 receptor (IL-4R) signaling plays a pivotal role in type 2 immune responses. It has been shown that IL-4 abrogates Th17 cell–mediated inflammation by selective silencing of IL-23 in APCs (35). Considering the crucial role of IL-17 in nasal clearance of *B*. *pertussis* in adult mice (27), we examined the involvement of IL-4R signaling in aP-caused prolonged nasal *B*. *pertussis* carriage. We found that maternal aP vaccination leads to increased concentrations of IL-4 and IL-13 in the blood of their offspring 5 days after challenge compared with the controls (Figure 6A), suggesting that maternal aP immunization enhances Th2 immune responses in the offspring. However, the concentrations were very low, and this prevented us from determining the source of these cytokines. To determine the role of IL-4R–dependent signaling in delayed nasal clearance of aP-immunized mice, we compared the bacterial loads in lungs and noses of aP-vaccinated adult control BALB/c mice with those of aP-vaccinated IL-4Rα–deficient BALB/c mice. aP vaccination resulted in lung clearance of both mouse strains, with slightly faster clearance kinetics in the IL-4Rα–deficient mice than in the BALB/c mice (Figure 6B). In contrast, and as shown previously (27), aP vaccination of adult BALB/c mice caused persistent nasal carriage of *B*. *pertussis*, while the aP-induced prolonged nasal carriage was abrogated in the IL-4Rα–deficient adult mice (Figure 6B). In neonatal IL-4Rα–KO mice, lung colonization was similar to neonatal BALB/c mice, and aP vaccination resulted in significant protection in both mouse strains (Figure 1, B and C, and Figure 6C). However, while maternal aP vaccination of BALB/c mice did not protect the offspring against nasal colonization by *B*. *pertussis*, but caused prolonged nasal carriage (Figure 1, B and C), it provided significant protection against nasal carriage in IL-4Rα–deficient mice (Figure 6C). To determine whether this was reflected by the role of IL-4 signaling on the T cell populations in the noses of aP-vaccinated versus nonvaccinated mice, we measured Trm cells in the nasal tissue 28 dpc with B1917. As shown in Figure 6D, aP immunization did not impair T cell recruitment in the noses of IL-4Rα^–/–^ mice. However, IL-17 production was still somewhat reduced but not as drastically as in BALB/c mice. Nevertheless, this IL-17 production was sufficient for the recruitment of neutrophils, which was not impaired by aP vaccination in the IL-4Rα^–/–^ mice, nor was the recruitment of APCs (Figure 6D). These results indicate that prolonged nasal carriage of *B*. *pertussis* in aP-immunized BALB/c mice and their offspring is mediated by the IL-4R signaling pathway.

### Influence of maternal pertussis immunization persists through adulthood.

**Figure 7:**
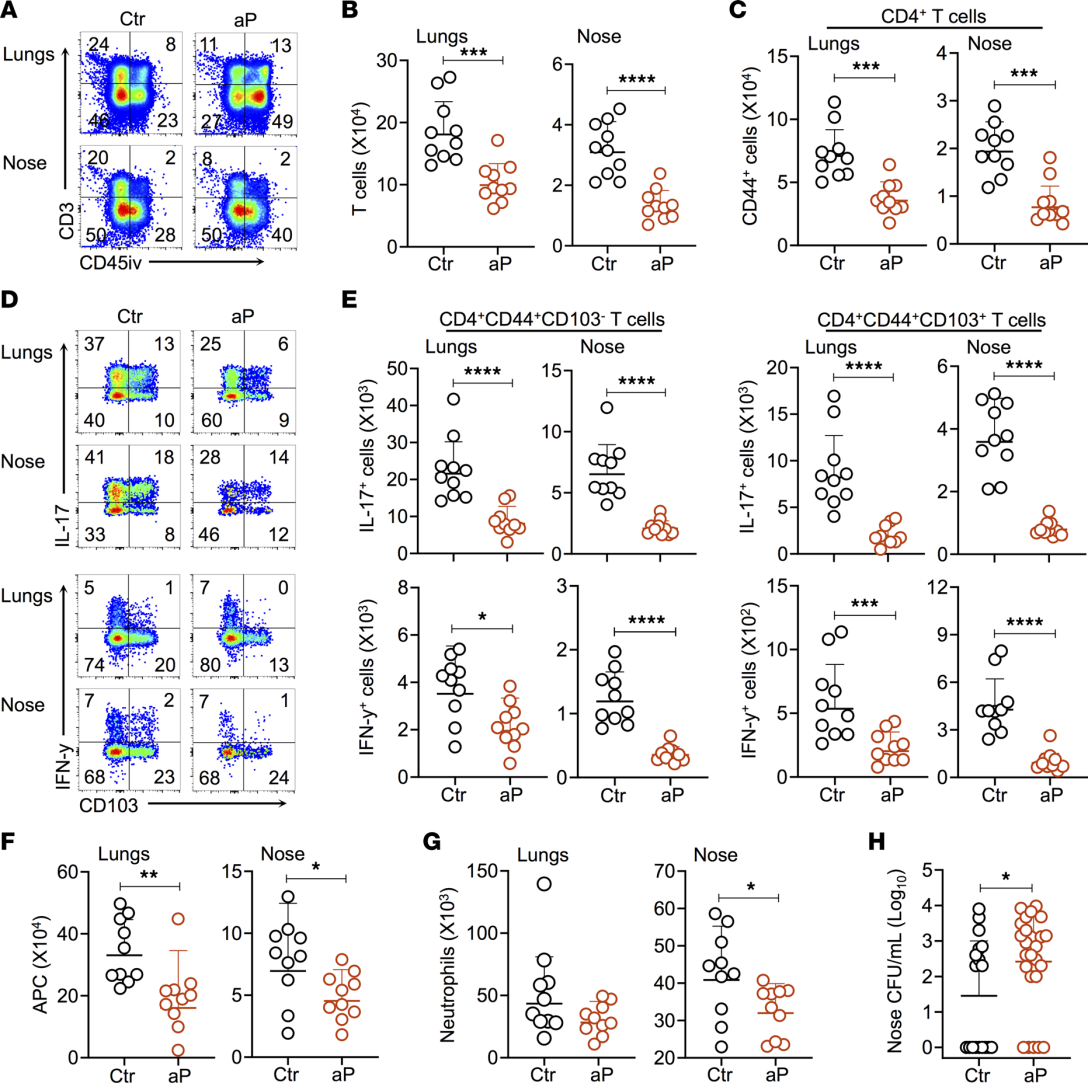
Long-term effect of maternal aP immunization on offspring T cell responses. Ten-week-old mice born to control (Ctr) or aP-immunized mice (aP) were infected with 1 ***×*** 10^6^ CFU B1917 and sacrificed 28 dpc. Ten minutes before euthanasia, mice were i.v. injected with anti–CD45-PE (CD45iv) antibodies. (**A**) Representative plots showing the recruitment of CD3^+^ T cells in the lungs and noses of aP and Ctr offspring. (**B**) Absolute numbers of T cells in the lungs (left panel) and noses (right panel) of aP and Ctr offspring. (**C**) Absolute numbers of CD44^+^CD4^+^ T cells in the lungs (left panel) and noses (right panel) of aP and Ctr offspring. (**D**) Representative plots showing the expression of IL-17 (upper panel) and IFN-γ (lower panel) by CD103^+^ T cells upon stimulation with PMA and ionomycin. (**E**) Absolute numbers of lung and noses CD4^+^CD44^+^CD103^–^ (left panels) and CD4^+^CD44^+^CD103^+^ T cells (right panels) expressing IL-17 (upper panels) or IFN-γ (lower panels). (**F**) Absolute numbers of APC in the lungs (left panel) and noses (right panel) of Ctr and aP offspring. (**G**) Absolute numbers of neutrophils in the lungs (left panel) and noses (right panel) of Ctr and aP offspring. (**H**) CFU counts on nose homogenates 56 dpc of Ctr and aP offspring. Results shown are geometric means ± SD. *n* = 4–10. Mann-Whitney tests were performed to compare Ctr and aP. *P < 0.05; **P < 0.01; ***P < 0.005; ****P < 0.001.

To determine whether the effect of maternal aP immunization on the T cell responses and prolonged nasal *B*. *pertussis* carriage in the offspring is transient or persists into adulthood, mice born to aP-immunized mothers or to control mice were infected with 1 ***×*** 10^6^ CFU of *B*. *pertussis* when they were 10 weeks old, and cellular immune responses in the lungs and noses were assessed 28 dpc. In these adult mice, T cell recruitment was still impaired in both lungs and noses by maternal aP vaccination (Figure 7, A and B). In particular, total numbers of CD4^+^CD44^+^ Th cells were significantly reduced in the aP mice compared with the controls (Figure 7C). This was also reflected by the reduced IL-17 and IFN-γ responses by the CD4^+^CD44^+^CD103^–^ and CD4^+^CD44^+^CD103^+^ Trm cells (more than 2-fold) in the aP mice (Figure 7, D and E). Impaired cellular immunity in these aP mice was also associated with diminished numbers of phagocytic cells, including APC and nasal neutrophils (Figure 7, F and G). Importantly, higher amounts of *B*. *pertussis* persisted in the noses of the aP mice than of the controls 56 dpc (Figure 7H).

### Influence of maternal aP vaccination is specific for B. pertussis.

**Figure 8:**
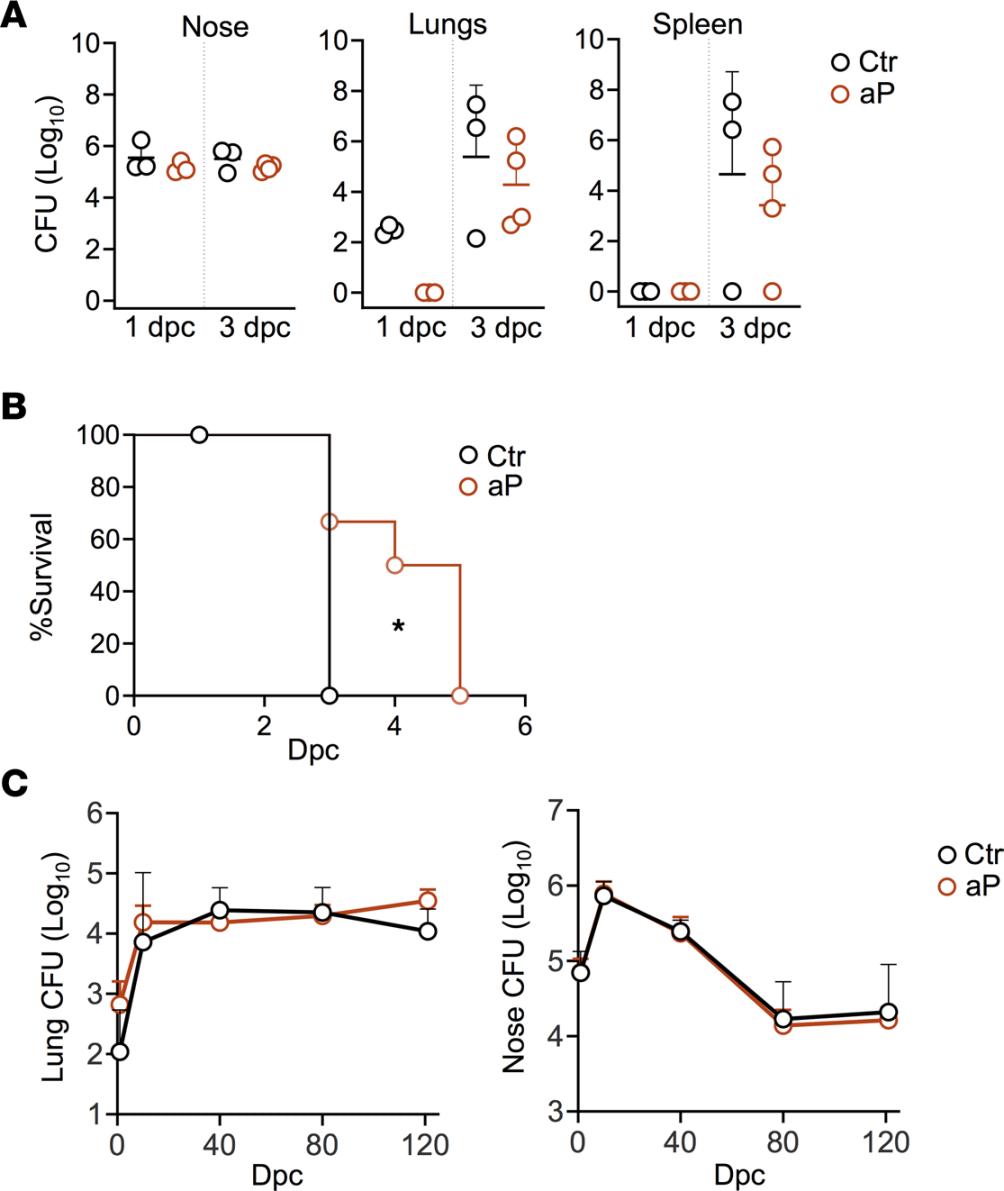
Effect of maternal aP immunization on colonization by *K*. *pneumoniae* and *S*. *pneumoniae* in the offspring. Neonatal mice born to control (Ctr) or aP-vaccinated mothers (aP) were i.n. infected with *K*. *pneumoniae* (**A** and **B**) or *S*. *pneumoniae* (**C**) 7 days after birth. One and 3 dpc with 50 CFU, *K*. *pneumoniae* CFU numbers were measured in nose, lungs, and spleen (**A**) (*n* = 3–4), and survival was monitored over time after *K*. *pneumoniae* infection (**B**) (*n* = 6). *S*. *pneumoniae* CFU numbers were measured in the lungs and noses (**C**) at indicated time points after challenge with 2 × 10^3^ CFU *S*. *pneumoniae* (*n* = 6–8). *P < 0.05.

To investigate whether maternal aP vaccination may also impact bacterial clearance of unrelated pathogens, we tested the effect of maternal aP immunization on colonization of the pups with a Gram-positive, *Streptococcus pneumoniae*, and a Gram-negative, *Klebsiella pneumoniae*, organism. Since *K*. *pneumoniae* infection is lethal for mice, we could only measure bacterial counts during the first days after infection. One and 3 dpc CFU counts in the nasal tissues, lungs, and spleen were similar between pups born from aP-vaccinated and unvaccinated mothers, although pups born to aP-vaccinated mice tended to have a lower bacterial burden in the lungs and spleens compared with the control mice (Figure 8A). However, this did not reach statistical significance. When survival was measured, pups from aP-vaccinated mice appeared to survive slightly but significantly longer than the control mice (Figure 8B). Maternal aP vaccination did also not hamper clearance of *S*. *pneumoniae* in the lungs or nasal tissues of the offspring, since up to 120 dpc the bacterial load in both organs were comparable between the 2 groups (Figure 8C)

## Discussion

Maternal immunization has proven an effective way to protect neonates against severe infectious diseases, including pertussis, which nevertheless remains one of the most poorly controlled vaccine-preventable diseases across the world. However, the effect of maternal immunization on the immune responses, especially the T cell responses, in the offspring is still poorly understood. Using a nonlethal mouse model of neonatal pertussis, we show here that maternal aP immunization impairs mucosal immunity against *B*. *pertussis* in the offspring and prevents natural *B*. *pertussis* clearance from the nasal tissue. The effect on delayed bacterial clearance in the nasal tissues is specific for *B*. *pertussis*, since maternal aP vaccination did not significantly prevent nasal clearance of their offspring by unrelated Gram-positive and Gram-negative pathogens, such as *S*. *pneumoniae* and *K*. *pneumoniae*, respectively. In fact, maternal aP vaccination appeared to slightly prolong survival of the pups upon infection with *K*. *pneumoniae*. We can therefore not exclude some degree of nonspecific bystander effect of maternal aP vaccination.

The aP-mediated inhibition of *B*. *pertussis* clearance in the nasal tissues was independent of the sex of the mice and affected both male and female mice to a similar extend. It was paralleled by decreased numbers of IL-17– and/or IFN-γ–producing parenchymal T cells in nose and lungs of *B*. *pertussis*–infected pups born to aP-vaccinated compared with nonvaccinated mothers. Furthermore, both CD11a and CD44 marker expression was also altered on T cells of pups born to aP-immunized mice. Lowered expression of these 2 markers in peripheral lymphoid organs might account for diminished recruitment of T cells at the site of infection, the lack of T cell activation (36), and lower Th1/Th17 differentiation (37, 38).

IL-17–producing CD4^+^ Trm cells responses are required to efficiently clear *B*. *pertussis* from the upper respiratory tract (27, 31). We have previously shown that aP vaccination inhibits the natural recruitment of these cells into the nasal tissue, which in turn impedes neutrophil recruitment and thereby prevents natural clearance of *B*. *pertussis* in the nose of adult mice (27). Here, we also provide evidence that aP vaccination alters cell-mediated immune responses to *B*. *pertussis* infection in an IL-4R–dependent manner. This was the case for both aP-vaccinated adult mice and their offspring. IL-4 has been shown to inhibit Th17 differentiation and IL-17 expression by silencing IL-23 in APC in adult mice (35). IL-4 signaling also enhances anti-CD3–induced Tbet and IFN-γ expression in both CD4^+^ and CD8^+^ T cells (39), and this may prevent the natural induction of Th17 cells. Type 2 cytokines, such as IL-4 and IL-13, and their signaling via IL-4Rs on neutrophils have also been linked to inhibition of several neutrophil effector functions (40). Signaling via IL-4Rs directly curtails neutrophil chemotaxis toward potent intermediary chemoattractants, inhibits the formation of neutrophil extracellular traps, and antagonizes the effects of granulocyte colony-stimulating factor on neutrophils. Consistently, we found that maternal aP immunization augments IL-4 and IL-13 concentrations in the serum of the offspring and alters neutrophil numbers and function in their nasal mucosa.

It is currently unknown which component of aP is responsible for the observed effects. It could be due to pharmacological effects of one or more of the vaccine antigens, to aluminium hydroxide used as adjuvant, or to the formaldehyde and/or glutaraldehyde treatment of some of the vaccine components. Future studies may be able to address this important point, if an agreement can be found with vaccine manufactures to make individual compounds of aP available for such studies.

The effects of maternal aP immunization on nasal carriage of *B*. *pertussis* in the offspring is long lasting and persists into adulthood. In addition, maternal aP immunization alters mucosal immunity in pups born long (>2 months) after the mother received the booster dose. Although not directly addressed here, this is likely due to maternal microchimerism (MMc), defined by the presence of maternal cells in the organs of the fetus, rather than epigenetic mechanisms that are mediated by unique signals experienced at specific developmental windows (41). MMc cells can be acquired during pregnancy and/or lactation and persist until adulthood (42). Consistent with this notion, persistent carriage of *B*. *pertussis* was observed after switching pups born to control (Ctr/aP) or aP-immunized mice (aP/Ctr) immediately after birth in both groups of foster pups, suggesting that both breastfeeding and in utero imprinting influence the pups’ mucosal cellular immune responses to *B*. *pertussis* infection.

The mechanism responsible for the transfer of cellular immunity from vaccinated mothers to their offspring via breastfeeding can involve uptake of APCs and/or mature T cells by the pups and their migration into the thymus, as has been shown in several studies (43–45). Recycling cells express the memory/migration marker CD44 (46), are involved in positive selection, and play a crucial role in training of the neonate immune system (47).

Alternatively, maternal IgG may also play a role in offspring immune modulation. IgG can interact directly with the offspring’s immune system, even in the absence of antigens (48). The transfer of maternal antigen-specific IgG influences antigen-specific immune responses later in the life by altering the repertoires of both T and B lymphocytes in the progeny (49). Although systemic infant CD4^+^ T cell responses are mostly unaffected by maternal antibody interference (50, 51), maternal antibodies have been shown to dampen mucosal T cell responses against commensal bacteria in mice (52) and limit the expansion of follicular Th cells in the germinal center (50).

It is not known which component of the breast milk or transplacental blood is responsible for the effects on offspring T cells. Both may contain aP-induced antibodies as well as cytokines, antigens, and other components, including immune cells. The observed long-lasting impact of maternal aP immunization on the mucosal immune responses of the offspring to *B*. *pertussis* infection argues in favor of maternally derived memory cells playing a role rather than antibodies induced by aP, consistent with our inability to correlate serum antibody levels with nasal carriage (not shown). Alternatively, we cannot rule out that the effect of maternal immunization on the nasal colonization of the switched pups may be due to differences in the maternal microbiome transferred to the pups, rather than breastfeeding itself. In any event, our observations indicate that the effect of maternal aP vaccination on nasal carriage in the offspring occurs in at least 2 ways.

Although the findings reported here are limited to mice, a recent study has shown that, in humans, maternal aP vaccination diminishes T cell responses to the primary series of pertussis vaccination in both term and preterm babies (53). However, the effect of maternal immunization on local Trm cells could not be investigated in these young babies, and the effect of maternal vaccination on nasal *B*. *pertussis* carriage in the offspring remains to be investigated in humans. Nevertheless, in a nonhuman primate model, maternal aP vaccination, although protective against pertussis disease in the pups, also resulted in prolonged nasal *B*. *pertussis* carriage in the offspring (54).Several studies suggest that, once the cellular immunity is polarized to a Th2 or Th1/Th17 response, it may be difficult to be reverted (55–57). Considering the importance of Th1/Th17 type immune responses for protection against *B*. *pertussis* infection, particularly in the nasal cavity, early-life Th2 skewing by current maternal aP immunization may, therefore, have a long-lasting negative impact on the control of pertussis, and alternative approaches to protect newborns from pertussis warrant consideration. Such alternative approaches may require the development of novel vaccines for maternal or neonatal vaccination. One such vaccine candidate, which was found to induce strong mucosal IL-17– and IFN-γ–producing Trm cell responses (58), is the live attenuated BPZE1 vaccine, currently in advanced clinical development (59). However, until this vaccine can be safely administered to neonates or pregnant women, maternal aP immunization remains the most effective strategy to protect neonates against pertussis in its most severe form in the first months of life (60).

## Methods

### Animals.

BALB/c mice were purchased from Charles Rivers Laboratory. IL-4Rα^–/–^ mice on a BALB/c background (61) were obtained from The Jackson Laboratory (stock no. 003514).

### Bacterial strain and growth conditions.

*B*. *pertussis* B1917 (62) came from the RIVM collection. For counter selection purposes, B1917 was electroporated with the pFUS2-BctA1 suicide plasmid to acquire gentamicin resistance as described (63), thereby yielding B1917GR. After electroporation, gentamicin-resistant derivatives were checked by PCR to verify the site of insertion of the pFUS2-BctA1 suicide vector in the bacterial genome. Bacteria were cultured at 37°C on Bordet-Gengou (BG) agar (Difco), supplemented with 1% glycerol, 10% defibrinated sheep blood, and 10 μg/mL gentamycin, as described (64). After 40 hours of growth, the bacteria were harvested by scraping the plates and resuspended in PBS at the density of 5 × 10^5^ CFU/mL for infection of neonatal mice, or 5 × 10^7^ CFU/mL for infection of adult mice. Whole–*B*. *pertussis* cell lysates were prepared and used for antibody determination as described (65). *S*. *pneumoniae* serotype 1 (clinical isolate E1586) was obtained from the National Reference Laboratory, Ministry of Health, Montevideo, Uruguay, and was grown statically at 37°C with 5% CO_2_ for 4–6 hours in Todd-Hewitt yeast broth, consisting Todd-Hewitt broth (Sigma-Aldrich) and 0.5% yeast extract (Becton Dickinson) as described (66). *K*. *pneumoniae* (ATCC 43816) was grown at 37°C in tryptic soy broth (Sigma-Aldrich) until mid-log phase.

### Protection experiments.

Adult BALB/c mice were immunized s.c. with 1/10 human dose of Infanrix (aP). Control mice received PBS instead. Mice were mated 28 days after priming. Mice were either boosted on the day of the mating (preconception boost) or 10 days afterward (maternal boost). Five- to 9-day-old neonates born to control or aP-immunized mice were infected nasally with 5 × 10^3^ CFU B1917GR, and adult BALB/c or IL-4Rα^–/–^ mice were infected nasally with 1 ***×*** 10^6^ CFU B1917GR as described (64). Alternatively, neonatal mice were infected with 50 CFU *K*. *pneumoniae* or with 2 × 10^3^ CFU *S*. *pneumoniae*. Placental and breast milk–derived protection against colonization in the offspring was evaluated by switching offspring from control (Ctr) and aP-vaccinated mothers within 12 hours after birth. Control pups nursed by aP-immunized mice (Ctr/aP) and pups from aP-vaccinated mothers nursed by control dams (aP/Ctr) were infected nasally with 5 × 10^3^ CFU B1917.

Mice were euthanized at different times after challenge, and lungs and noses were harvested and homogenized for CFU measurement by plating 10-fold serial dilutions onto BG agar plates containing 10 μg/mL gentamicin. Lungs were harvested as described (58), and nose extracts were prepared as follows. Euthanized mice were held on their backs. After pulling off the nostril skin with tweezers, an incision was made on each side of the head between the nostrils and the cheeks and following the side of the head up to the neck to remove the skin from the top of the head. Then, an incision was made on each side of the head to cut and remove the lower jaw. Finally, incisions at the base of the palate and on each side of the head were made to remove the part of the head containing the nasal cavities. Residual skin, eyeball, and cheek muscle were carefully removed from the recovered tissues. The remaining material was then minced into a homogenous paste with a scalpel on a dish plate and treated with 1 mL of an enzyme cocktail containing collagenase D (0.6 mg/mL; Roche) and DNase I (20 U/mL; Sigma-Aldrich) for tissue dissociation.

### Antibody titer determination.

To determine antibody titers in the sera, standard ELISA protocols were used. Briefly, 96-well plates (Nunc) were coated overnight with 1 μg per well of total B1917 lysate and then blocked for 2 hours at room temperature with 2% BSA (MilliporeSigma) in PBS (Thermo Fisher Scientific)/0.5% Tween (MilliporeSigma). B1917 lysates were prepared from a 50 mL bacterial culture grown in Stainer Scholte medium until the OD_600_ reached 0.8. The bacteria were then harvested by centrifugation (15 minutes at 4°C and 15,000*g*), resuspended in 5 mL PBS with a cocktail of antiproteases complete mini (Roche) and 10 μg/mL DNaseI (Roche) and lysed with the FastPrep-24 (MP Biomedicals [MPbio]) tissue and cell homogenizer. Bacteria (1 mL) were lysed in lysing matrix B 2 mL tubes containing 0.1 mm silica spheres (MPbio) by performing 2 beating cycles at 6 m/s for 45 seconds. Bacteria were incubated on ice prior to disruption and after each beating cycle. The protein concentration was quantified using BCA test (Thermo Fisher Scientific) following the manufacturer’s instructions. The coated plates were incubated for 2 hours at 37°C with serum samples in 2-fold serial dilutions in PBS/0.5% Tween. After 3 washes with PBS/0.5% Tween, horseradish peroxidase–goat (HRP-goat) anti–mouse IgG (1:6,000) (MilliporeSigma, A-0168) was added and the plates were incubated at 37°C for 1 hour. After washing 5 times with PBS/0.5% Tween, the reaction was developed by using TMB (Interchim) and 1M H_3_PO_4_, and the plates were read using BioTek ELx8000.

### Neutrophil depletion.

Anti–Ly-6G antibody (Bio X Cell, clone 1A8) was used to deplete the neutrophils of infected C57BL/6 mice. Mice received 175 μg of the 1A8 antibody i.v., and 25 μg were administered i.n. 1 day prior to infection; 350 μg were administered i.v. and 50 μg i.n. every 2 dpc. Control mice received the corresponding isotype control antibody (clone 2A3; Bio X Cell). Three and 14 dpc, the mice were sacrificed and the noses were harvested, homogenized, and plated onto Bordet Gengou blood agar plates to count CFUs.

### Cytometric bead array.

Amounts of IL-4 and IL-13 were determined using a cytometric bead array (CBA) mouse enhanced sensitivity kit (Becton Dickinson). The CBA Enhanced Sensitivity Flex Set system was applied to detect small amounts of cytokines in the serum. Experiments were carried out following the manufacture’s instructions. In brief, 20 μL of each blood sample was incubated for 2 hours with appropriate amounts of detection beads, which were specific for each investigated cytokine. Afterward, samples were incubated for 2 hours with detection reagent, followed by addition of 100 μL of Mouse Enhanced Sensitivity Detection Reagent (Becton Dickinson). After incubation for 1 hour at room temperature, wells were washed, and content was transferred to 1.5 mL microcentrifuge tubes for analysis on an Attune NxT using the Attune NxT Software (Thermo Fisher Scientific) and analyzed using the FlowJo Software (v10, Tree Star Inc.). The amount of cytokine was calculated using a specific standard curve (in fg/mL).

### Cell isolation for flow cytometry analysis.

Ten minutes before euthanasia, mice were injected i.v. with 10 μg of anti–CD45-PE antibody (eBioscience, 30-F11, 12-0451-82) to allow for the distinction of circulating T cells (CD45-PE^+^) and resident T cells or infiltrated immune cells (CD45-PE^−^). The spleen was then minced into a homogenous paste with a scalpel on a dish plate and then treated with 1 mL of an enzyme cocktail containing collagenase D (0.6 mg/mL; Roche) and DNase I (20 U/mL; Sigma-Aldrich). The supernatant and tissue from the culture dish were passed through a cell strainer by gently pressing in a circular motion with the flat end a fresh piston/plunger from a sterile 3 cc syringe to obtain a single-cell suspension. RBCs were lysed using ACK lysing buffer (Thermo Fisher Scientific). Isolated cells were stimulated with 50 ng/mL PMA (InVivoGen) and 500 ng/mL ionomycin (MilliporeSigma) in the presence of 5 μg/mL brefeldin A (MilliporeSigma) for 4 hours at 37°C. Cells were incubated with Dead Cell Stain Kit LIVE/DEAD Aqua (Invitrogen) first, as recommended by the supplier, and were then incubated with Fc block (BD Biosciences, dilution 1:50), followed by surface staining with the following fluorochrome-conjugated antibodies for up to 13-parameter flow cytometric analysis: CD45-APC (30-F11, 559864), CD69-FITC (H1.2F3, 11-0691-82), CD8-AF700 (53-6.7, 56-0081-82), and CD3–APC-eF780 (17A2, 47-0032-82) from eBioscience; CD44-BV605 (IM7, 103047), CD4-BV785 (RM4-5, 139314), CD64–PE-Cy7 (X54-5/7.1, 100552), CD11c-BV711 (N418, 139314), CD24-PB (M169, 101820), CD86-AF700 (GL-1, 105024), CD66a-FITC (Mab-CC1, 134518), and CD103–PerCP-Cy5 (2E7, 121416) from BioLegend; CD103–PE-CF594 (M290, 565849) and Ly-6G–PE-CF594 (1A8, 562700) from BD Biosciences; and ly6c1–PE-Cy5.5 (Monts 1,LS-C811215-50) from LSbio. For the detection of intracellular cytokines, cells were fixed and permeabilized using a FoxP3 transcription factor staining buffer set (eBioscience), incubated with normal rat serum (eBioscience) (1:50), and stained with the following antibodies: IL-17–V450 (TC11-18H10, 560522, dilution 1:200) from BD Biosciences and IFN-γ–PE-Cy7 (XMG1.2, 25-7319-41, dilution 1:400) from eBioscience. Fluorescence minus one sample were used as controls. The gating strategy for the identification of antigen presenting cells is given in Supplemental Figure 5. FACS samples were acquired on an Attune NxT using the Attune NxT Software (Thermo Fisher Scientific) and analyzed using the FlowJo Software (v10, Tree Star Inc.). Since the Attune NxT cytometer measures the volume of the acquired sample, the total number of cells was calculated by multiplying cell concentrations by total volume of samples.

### Statistics.

Statistical analyses were performed by nonparametric Mann–Whitney *U* tests using the GraphPad Prism software. *P* < 0.05 was considered significant.

### Study approval.

All animal experiments were carried out in accordance with the guidelines of the French Ministry of Research, and the protocols were approved by the Ethical Committees of the Region Nord Pas de Calais and the Ministry of Research (agreement APAFIS # 2019052015506229 V4).

### Data availability.

Data will be made available by the corresponding author upon reasonable request. Values for all data points in graphs are reported in the Supporting Data Values file.

## Author contributions

Conceptualization was contributed by VD and CL. Methodology was contributed by VD and JC. Investigation was contributed by VD, JC, MD, and CL. Funding acquisition was contributed by CL. Supervision was contributed by CL. Writing of the original draft was contributed by VD. Review and editing of the manuscript were contributed by VD and CL.

## Supplementary Material

Supplemental data

Supporting data values

## Version 1 08/15/2023

In-Press Preview

## Version 2 09/22/2023

Electronic publication
