# Supplementary material for: Maternal acellular pertussis vaccination in mice impairs cellular immunity to Bordetella pertussis infection in offspring
Source: JCI Insight. 2023 Sep 22;8(18):e167210. doi: 10.1172/jci.insight.167210 (PMC10561720; doi:10.1172/jci.insight.167210)
Supplement: Supplemental data [file jciinsight-8-167210-s017.pdf]

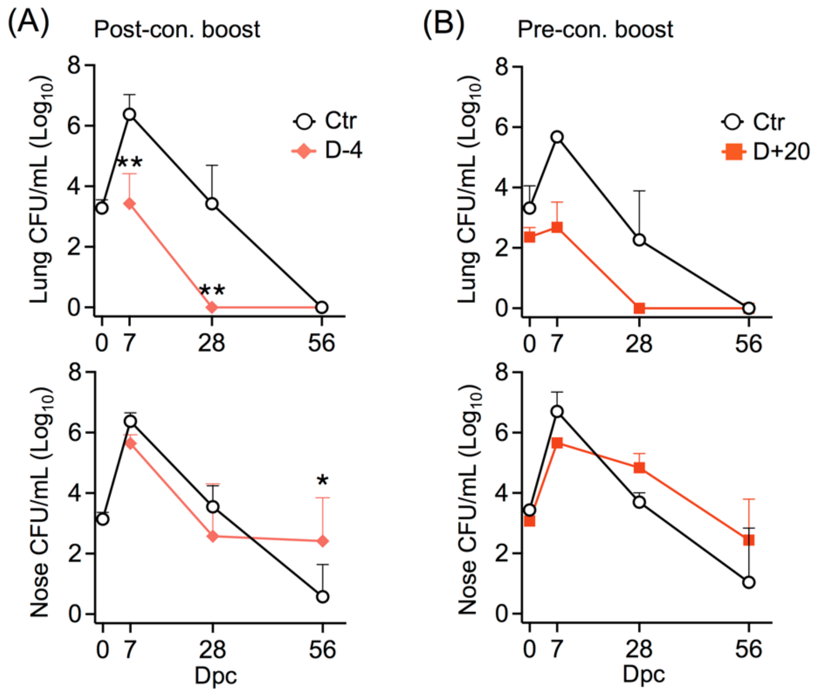

Figure S1. Effect of maternal aP immunization schedules on lung and nasal colonization in the infected offspring. Adult (8 week-old) female BALB/c mice were immunized subcutaneously with 1/10 human dose of aP. Control mice (Ctr) received PBS instead. Mice were either boosted 10 days after mating (A, post-con. boost), or on the day of mating (B, pre-con. boost). Five to nine days after birth the pups were nasally infected with $5x10^3$ CFU B1917. Lungs and noses were harvested at indicated time points after challenge for CFU counting in the lungs (upper panels) and noses (lower panels). Results shown are geometric means ± SD. n=3-11 for the Ctr groups and n= 3-10 for the aP groups. Mann-Whitney tests were performed to compare Ctr and aP offspring. *, $p < 0.05$; **, $p < 0.01$.

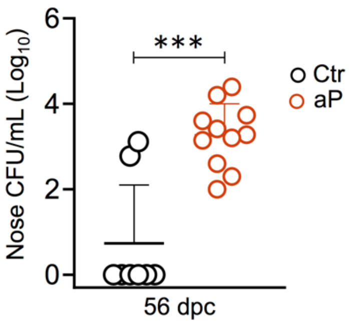

Figure S2. Long-lasting effects of maternal aP immunization on nasal *B. pertussis* carriage in offspring. Adult female BALB/c mice were vaccinated with aP and boosted 28 days later, 2.5 month before conception. The pups were infected 6 to 9 days after birth with $5x10^3$ CFU B1917 and sacrificed 56 dpc to assess the bacterial burden in the noses of aP and control (Ctr) mice; n= 8-11. Mann-Whitney tests were performed to compare Ctr and aP offspring. ***, $p < 0.005$.

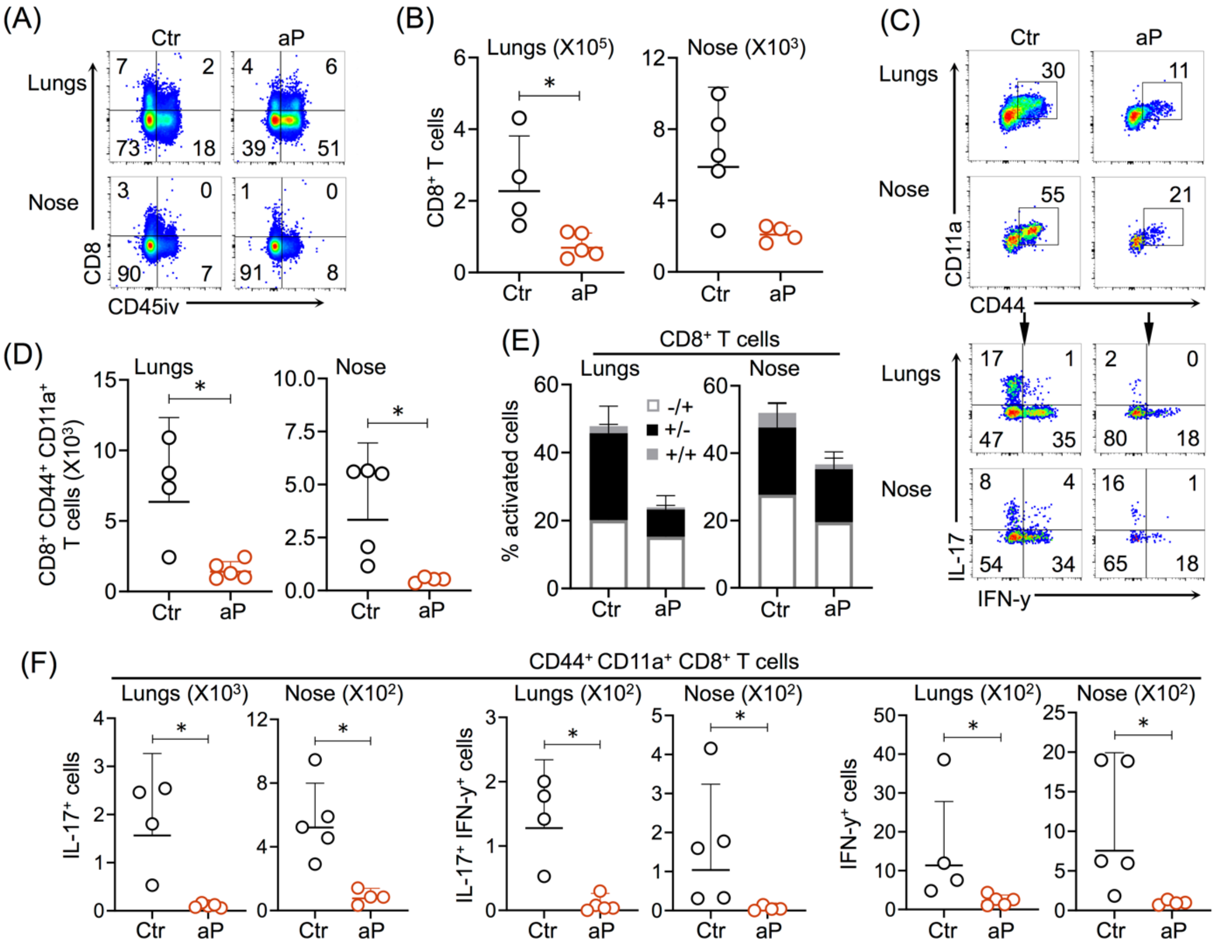

Figure S3. Effect of pre-conception aP immunization on recruitment and activation of cytotoxic T cells in the lower and upper respiratory tracts of the offspring. Neonatal mice were infected with 5.10^3 CFU B1917 and sacrificed 28 dpc. Ten minutes before euthanasia, mice were intravenously injected with anti-CD45-PE (CD45iv) antibody. (A) Representative plot showing the recruitment of $CD8^+CD45iv^-$ T cells in the lungs (upper panels) and nose (lower panels) of aP (right panels) and Ctr (left panels) offspring. (B) Absolute numbers of $CD8^+CD45iv^-$ T cells in the lungs (left panel) and nose (right panel) of offspring. (C) Representative plots showing the expression of CD11a and CD44 on $CD8^+$ T cells (upper panel) and expression of IL-17 and IFN-γ upon stimulation with PMA and ionomycin (lower panel). (D) Absolute numbers of pulmonary (left panel) and nasal $CD8^+$ T cells (right panel) expressing CD44 and CD11a. (E) Percentages of activated $CD8^+$ T cells in the lungs (left panel) and noses (right panel) producing IL-17 (black), IFN-γ (white) or both (light grey). (F) Absolute numbers of $CD44^+CD11a^+CD8^+$ T cells expressing IL-17 (left panels), IFN-γ (right panels) or both (middle panels). Results shown are geometric means ± SD. $n = 4\text{-}5$. Mann-Whitney tests were performed to compare Ctr and aP offspring. *, $p < 0.05$.

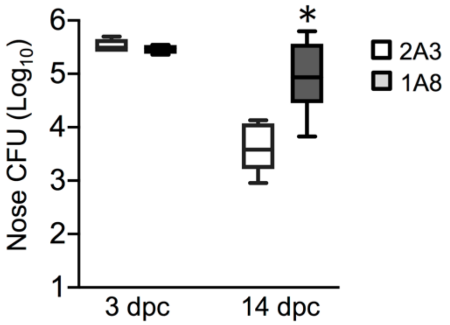

Figure S4. Effect of neutrophil depletion on nasal colonization by *B. pertussis*. C57BL/6 mice were treated with anti-Ly-6G antibodies (1A8) or isotype control antibodies (2A3) one day prior and every two days after infection with *B. pertussis*. Bacterial burden in the noses was measured 3 and 14 dpc (n=4-5). *, $p < 0.05$.

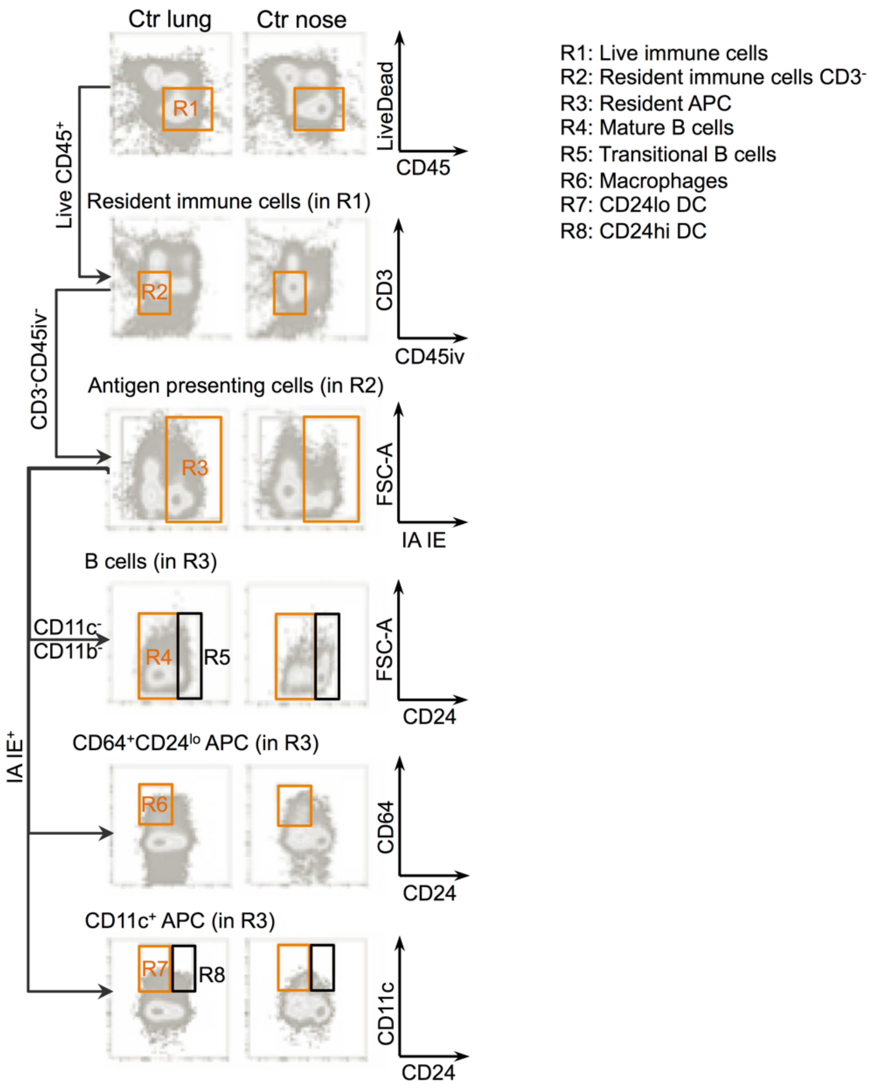

Figure S5. Gating strategy for the identification of antigen presenting cell subtypes. Ten minutes before euthanasia, mice are injected intravenously with anti-CD45-PE (CD45iv) antibody. Antigen-presenting cells in lungs (left panels) and noses (right panels) expressed IAIE. Mature B cells are $CD3^{-}CD11c^{-}CD11b^{+}$ and down-regulated CD24 expression. Macrophages up-regulated CD64 and expressed low levels of CD24. Dendritic cells are $CD11c^{+}$ and can be divided into $CD24^{hi}$ and $CD24^{lo}$ dendritic cells.
